# Multi-drug resistant *Klebsiella*-induced corneal ulcer following pterygium surgery: a case report

**DOI:** 10.1186/s12886-023-02928-y

**Published:** 2023-04-24

**Authors:** Kenan Calisir, Hassan Muhumed Mohamed, Aisha Abdirahman Yussuf

**Affiliations:** Mogadishu Somalia Turkey Training and Research Hospital, Ophthalmology Department, Mogadishu, Somalia

**Keywords:** *Klebsiella* keratitis, Pterygium surgery, Multi-drug resistance, Case report

## Abstract

**Background:**

Management of pterygium is dependent on the grading of pterygium and its clinical presentation (inflamed or quiescent), and surgical excision is the final choice of treatment for the pterygium extending beyond the limbus. Infectious keratitis is one of the most commonly reported complications in recent years. To the best of our knowledge, *Klebsiella* keratitis after pterygium surgery has not been described in the current literature. Here, we report a patient with corneal ulcer formation following pterygium surgical excision.

**Case presentation:**

A 62-year-old woman presented with complaints of pain, blurred vision, photophobia and redness in her left eye for a month. She had a history of pterygium surgical excision two months ago. Slit-lamp examination showed conjunctival congestion, a central whitish corneal ulcer with a central epithelial defect, and hypopyon. Corneal scraped sample revealed multidrug resistant (MDR) *Klebsiella pneumonia* and the strain was found to be sensitive to cefoxitin and ciprofloxacin. Intracameral cefuroxime (1 mg/0.1 mL) injection, fortified cefuroxime ophthalmic suspension (50 mg/mL) and moxifloxacin ophthalmic suspension (0.5%) were successfully administered to control the infection. Since residual central stromal opacification remained persistent, final visual acuity did not improve beyond finger counting at two meters.

**Conclusions:**

*Klebsiella* keratitis is a rare and sight-threatening complication following pterygium excision. This report emphasizes the importance of close follow-up examination following pterygium surgeries.

## Background

Pterygium is a relatively common ocular surface disorder and surgical excision is remains the preferred treatment option. So as to decrease the rate of recurrence, excision is usually combined with a conjunctival autograft and/or mitomycin-C (MMC) administration. Infectious keratitis, corneal thinning, scleral melting, endophthalmitis, and peripheral ulcerative keratitis are commonly reported complications in recent years [1–4]. Bacterial keratitis can damage vision through permanent corneal scarring and even perforation, eventually causing the loss of an eye. If timely identified and optimal treatment is administered, considerable morbidity associated with severe microbial keratitis can be prevented.

Multidrug resistant (MDR) *Klebsiella pneumonia* is an opportunistic pathogen with a rising mortality rate, occasioned a wide range of community-acquired and nosocomial infections [5]. Although there are very few reports associated with *Klebsiella* induced keratitis following deep anterior lamellar keratoplasty (DALK) [6–8], contact lens-related keratitis [9], and post-corneal repair keratitis [10], no *Klebsiella* keratitis following pterygium removal has been reported. Here, we present a patient with *Klebsiella pneumonia* keratitis following pterygium surgery from Mogadishu, Somalia.

## Case presentation

A 62-year-old woman presented to the ophthalmology outpatient clinic with complaints of pain, blurred vision, photophobia, and redness in her left eye for a month. She had a history of pterygium surgical excision two months ago at another hospital in Mogadishu, Somalia. The patient had no history of ocular trauma and underlying systemic disorders such as diabetes mellitus. Patient was not informed whether topical MMC was used intraoperatively during her surgery. Patient was using fortified vancomycin ophthalmic suspension (50 mg/mL), fortified gentamicin ophthalmic suspension (14 mg/mL), atropine ophthalmic suspension, natamycin ophthalmic suspension (5%) and doxycyline capsule (100 mg). Despite these empirical treatment with ophthalmic suspensions and oral antibiotic, the patient was not responding to the treatment.

**Fig. 1:**
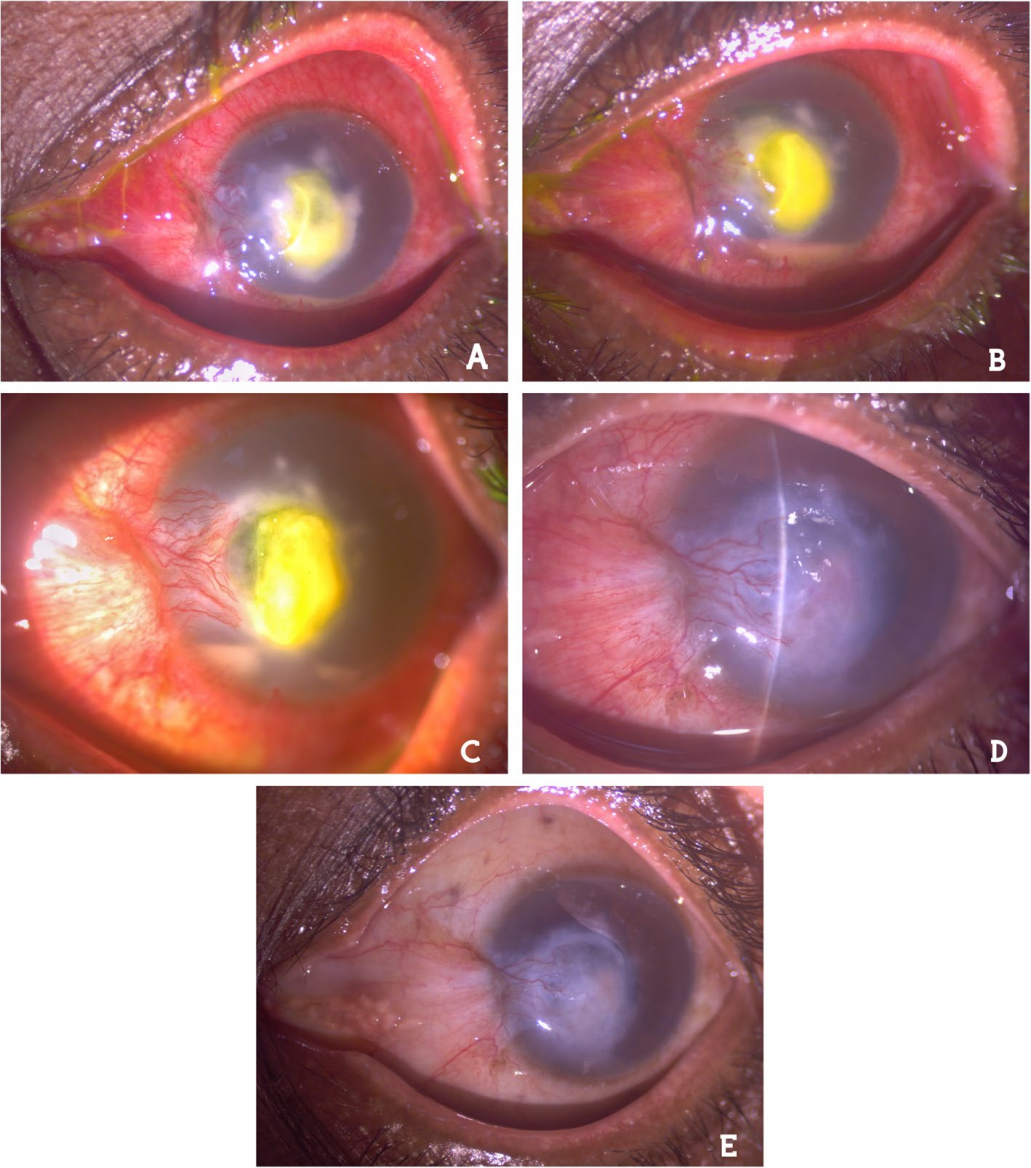
Slit-lamp examination. **a** Corneal ulcer with a central fluorescein stain and hypopyon. **b** Increased hypopyon level and persisting stromal ulcer. **c** Decreased hypopyon on the 3^rd^ day of intracameral cefuroxime injection. **d** The hypopyon was disappeared and the stromal infiltrates was regressing on the 7^th^ day of cefuroxime + moxifloxacin. **e** Residual central stromal opacification remained at 3^rd^ month

The visual acuity in the right eye was 20/20, while vision in the affected eye was restricted to perception of hand movement (HM). Slit-lamp examination showed severe conjunctival congestion, a central whitish corneal ulcer measuring about 6 × 6 mm with a central epithelial defect and unclear margin, and hypopyon (Fig. 1a). Pupil and lens were not visible due to the ulcer formation, and intraocular pressure was normal by digitally. We stopped the empirical treatment and performed corneal scraping under topical anesthesia for bacterial and fungal cultures a day later. The patient continued to use fortified vancomycin ophthalmic suspension, ceftazidime ophthalmic suspension (100 mg/mL) and voriconazole ophthalmic suspension (10 mg/mL) hourly during day, and cyclopentolate (1%) three times a day until the result of the microbiology report (7 days).

Gram stain of the scraping material did not reveal any specific organisms. Culture revealed gram-negative bacilli identified as *Klebsiella pneumonia* with conventional methods. The strain was found to be resistant to clindamycin, amikacin, vancomycin, quinupristin/dalfopristin, gentamicin, teicoplanin, ampicillin/sulbactam, fucidic acid, and cotrimaxasole, but sensitive to cefoxitin and ciprofloxacin. In vitro antibiotic susceptibility testing of the bacterial isolates was performed by Kirby- Bauer disc diffusion method [11] and interpreted in accordance with Clinical Laboratory Standards Institute criteria [12].

In the first week follow-up examination, there was no relief in the patient’s complaints despite the empirical antimicrobial treatment and hypopyon level was increased (Fig. 1b). All previous topical ophthalmic suspensions were stopped and intracameral cefuroxime (1 mg/0.1 mL) injection was performed under topical anesthesia in the operating room environment. Besides that topical fortified cefuroxime (50 mg/mL) and moxifloxacin (0.5%) hourly, cyclopentolate (1%) 3 times a day and oral ciprofloxacin (500 mg) twice a day were initiated. The hypopyon level began to decrease in 3 days (Fig. 1c). Therefore, fortified cefuroxime and moxifloxacin ophthalmic suspension doses were reduced to every two hourly administration. After 7 days; the hypopyon disappeared and the stromal infiltrates were steadily regressing (Fig. 1d), oral ciprofloxacin was stopped and the patient was asked to continue topical antibiotic ophthalmic suspensions 4 times a day, and cyclopentolate twice a day for 3 weeks more. At 4^th^ week; the conjunctival congestion and stromal infiltrates resolved. The patient felt comfortable and did not report any adverse and/or unanticipated events. Final visual acuity did not improve beyond finger counting at two meters due to residual central stromal opacification at 3^rd^ month (Fig. 1e).

## Discussion and conclusions

Excessive scraping, cauterization, adjunctive antiproliferative treatment such as MMC, and lack of experience in surgery were defined as remarkable risk factors for infectious sclerokeratitis in previous studies [1–3]. Since the patient had a pterygium surgery performed at another hospital, we do not have information about these possible risk factors. However, we do not have an accurate evidence regarding the original diagnosis leading to the conjunctival surgery, except for the patient’s statement. Keratitis after pterygium surgery is not common, in a retrospective chart-review study from Iran, Soleimani et al. reported five patients who developed keratitis following surgery out of a total of 2118 pterygium operations from January 2016 till December 2017 [2]. Their cultures predominantly demonstrated gram-positive bacteria contrary to our patient’s culture.

MDR gram-negative bacteria including *Klebsiella pneumonia* is emerging group of organisms related to nosocomial infection. Hospital-acquired *Klebsiella* can be found in the conjunctiva and sometimes cause severe ocular infection in patients during the hospital stay or afterwards [13]. The latency period between pterygium removal and keratoscleritis was reported as 10 days to 18 years in the previous reports [2, 14]. Our patient had no history of ocular trauma and/or underlying risk factors, moreover her complaints associated with keratitis started a month later following pterygium excision. Consequently, we may speculate that she had a corneal ulcer related to pterygium surgery.

*Klebsiella pneumonia* keratitis was reported to be treated with various topical antibiotic ophthalmic suspensions [1, 3, 6, 9]. In our case, antibiotic susceptibility profile of the isolates came positive for cefoxitin and ciprofloxacin. We preferred intracameral cefuroxime injection which belongs to the same antibiotic group with cefoxitin in order to control the infection as soon as possible.

*Klebsiella* keratitis after pterygium surgery may vary from small peripheral infiltrates to severe central ulcers with sight-threatening potential, as in our case [1]. Although the infection resolved, residual central stromal opacification remained persistent and final visual acuity did not improve very well. However, this was an acceptable outcome due to the fact that need for invasive procedures such as therapeutic penetrating keratoplasty or evisceration were eliminated and the patient can have a chance for elective keratoplasty in the future.

In conclusion, patients after pterygium surgery need a close follow-up. *Klebsiella* keratitis following pterygium surgical excision is a rare but sight-threatening complication. Early diagnosis and appropriate treatment can prevent need for invasive surgical and tectonic procedures to control the infection.

## Data Availability

Not applicable.

## Abbreviations

MMC: Mitomycin-C
MDR: Multidrug resistant
DALK: Deep anterior lamellar keratoplasty
